# Low Genetic Diversity in Wide-Spread Eurasian Liver Fluke *Opisthorchis felineus* Suggests Special Demographic History of This Trematode Species

**DOI:** 10.1371/journal.pone.0062453

**Published:** 2013-04-25

**Authors:** Ilja I. Brusentsov, Alexey V. Katokhin, Irina V. Brusentsova, Sergei V. Shekhovtsov, Sergei N. Borovikov, Grigoriy G. Goncharenko, Lyudmila A. Lider, Boris V. Romashov, Olga T. Rusinek, Samat K. Shibitov, Marat M. Suleymanov, Andrey V. Yevtushenko, Viatcheslav A. Mordvinov

**Affiliations:** 1 Laboratory of Molecular Mechanisms of Pathological Processes, Institute of Cytology and Genetics, Siberian Branch, Russian Academy of Sciences, Novosibirsk, Russia; 2 Laboratory of Molecular Biotechnology, Institute of Cytology and Genetics, Siberian Branch, Russian Academy of Sciences, Novosibirsk, Russia; 3 Department of Animal Biotechnology, S.Seifullin Kazakh Agrotechnical University, Astana, Republic of Kazakhstan; 4 Faculty of Biology, Francisk Skorina Gomel State University, Gomel, Republic of Belarus; 5 Department of Veterinary Medicine, S.Seifullin Kazakh Agrotechnical University, Astana, Republic of Kazakhstan; 6 Scientific Department, Voronezh State Biosphere Reserve, Voronezh, Russia; 7 Department of Parasitology, The Baikal Museum at the Irkutsk Scientific Center, Siberian Branch, Russian Academy of Sciences, Listvyanka, Irkutsk, Russia; 8 Department of Epizootological Problems, All-Russian K.I. Skryabin Institute of Helminthology, Moscow, Russia; 9 Department of Parasitology Ichthyopathology and Arachnology, National Scientific Center “Institute of Experimental and Clinical Veterinary Medicine”, Kharkov, Ukraine; CNRS, Université de Bourgogne, France

## Abstract

*Opisthorchis felineus* or Siberian liver fluke is a trematode parasite (Opisthorchiidae) that infects the hepato-biliary system of humans and other mammals. Despite its public health significance, this wide-spread Eurasian species is one of the most poorly studied human liver flukes and nothing is known about its population genetic structure and demographic history. In this paper, we attempt to fill this gap for the first time and to explore the genetic diversity in *O. felineus* populations from Eastern Europe (Ukraine, European part of Russia), Northern Asia (Siberia) and Central Asia (Northern Kazakhstan). Analysis of marker DNA fragments from *O. felineus* mitochondrial *cytochrome c oxidase subunit 1* and *3* (*cox1, cox3*) and nuclear rDNA *internal transcribed spacer 1* (*ITS1*) sequences revealed that genetic diversity is very low across the large geographic range of this species. Microevolutionary processes in populations of trematodes may well be influenced by their peculiar biology. Nevertheless, we suggest that lack of population genetics structure observed in *O. felineus* can be primarily explained by the Pleistocene glacial events and subsequent sudden population growth from a very limited group of founders. Rapid range expansion of *O. felineus* through Asian and European territories after severe bottleneck points to a high dispersal potential of this trematode species.

## Introduction

*O. felineus*, *O. viverrini* and *Clonorchis sinensis* are three species from the genus *Opisthorchis* (Opisthorchiidae, Trematoda) that are known to cause serious human diseases affecting bile ducts and the gall bladder. Opisthorchis infection is recognized as the major risk factor of cholangiocarcinoma [1]–[5]. An estimated 12.5, 67.3 and 601 million people are currently at risk for infection with *O. felineus*, *O. viverrini* and *C. sinensis*, respectively [6].

All species within the genus *Opisthorchis* are obligate endoparasites with a complex three-host life cycle (http://www.dpd.cdc.gov/dpdx/HTML/Opisthorchiasis.htm). The first intermediate hosts of *O. felineus* are sister-species of freshwater snails *Bithynia* (*B. leachi, B. troscheli, B. inflata*) [7]. Notably, while in the first intermediate host, the parasite undergoes extremely active asexual reproduction [7]. Fishes of the Cyprinidae family serve as the second intermediate hosts. Metacercaria, the encysted and resting stage of a fluke larva, accumulate in muscles of susceptible fish. Mature flukes live in the liver of definitive hosts (fish-eating mammals, including humans). Human infections occur by consuming raw or undercooked fish infested with metacercariae.

Among human-infecting members of the genus *Opisthorchis*, *O. felineus* has the largest distribution range, which does not overlap with the ranges of *O. viverrini* (found in Thailand, Lao PDR, Vietnam and Cambodia) [8] or *C. sinensis* (China, Korea, Lao PDR, Vietnam and The Russian Far East) [8]–[10]. The major range of *O. felineus* spans over the territory of the Ob-Irtysh River basin (West Siberia), the Volga-Kama River system, as well as the Don and the Dnieper watersheds (Eastern Europe). The Yenisei River basin delimits the eastern border of the areal [7]. Outside of Russia, high prevalence of *O. felineus* has been reported in Belarus [11] and Ukraine [12]. In 1962, Erhardt and colleagues reported *O. felineus* in the final hosts in most European countries [13]. Recently human and animal infections in the European Union have been reported in Germany [14]–[17], Italy [18]–[20], Portugal [21], Greece [22] and the Netherlands [23]. Yet, natural populations of *O. felineus* have only been described for Italy and Germany [14], [18]. Prevalence of human infestation with flukes ranges from 80% in certain regions of Siberia to sporadic cases in the Central and Southern Europe [7]. Infestation rate of fish-eating mammals in the natural foci in the Ob-Irtysh basin varies from 40 to 70%, and ranges 12–28% throughout the Dnieper River basin [7]. In Germany, 7–30% of foxes were reported to be infected [17], [24], whereas during the recent opisthorchiasis outbreaks in Italy, as much as 46.4% of cats were positive [18].

Over the past few years, many efforts focused on studying the population genetic structure and heterogeneity of important trematode species including *Schistosoma mansoni* [25]–[30], *S. japonicum* [31]–[33], *Paragonimus westermani* [34], [35], *Fasciola hepatica* [36]–[40], *F. magnum* [41] *Echinostoma* sp. [42], [43]. As a result, distinct species were molecularly identified, and genetic diversity within species has been estimated. Furthermore, plausible sites of origin and species expansion routes were defined. The majority of these studies relied upon the analysis of mitochondrial (mtDNA) and nuclear rDNA gene variation. As a molecular marker, mitochondrial DNA has several important advantages. Because of its elevated mutation rate, it is highly variable in natural populations. Thus it can be quite informative regarding short-term population history [44]–[46]. Additionally, mitochondrial DNA generally does not recombine [44], [47]. This is particularly useful for studies of intra-species variation. It should be noted that trematode worms are hermaphrodites, so every individual may contribute to the population mtDNA pool. Embedded in the nuclear ribosomal DNA regions, *internal transcribed spacer* sequences, *ITS*, evolve much faster than genes encoding ribosomal RNA [48]. Due to their high variability, *ITS* have been employed to resolve phylogenetic problems in closely related species and they are the markers of choice in taxonomic studies of digeneans [49].

Opisthorchiasis and clonorchiasis are diseases with pronounced impact on public health in the countries of South-Eastern and Eastern Asia. Two parasitic species that cause these diseases, *O. viverrini* and *C. sinensis*, have been the focus of active research over the past several years. In particular, this research involved studies of genetic variation in *O. viverrini* populations. Based on the sequences of several mitochondrial DNA markers (*NADH dehydrogenase subunit 1* gene (*nad1*) and *cox1*), genetic variation among different geographical isolates of *O. viverrini* from Thailand, Cambodia and Lao PDR has been detected [50], [51]. Using microsatellite DNA genetic markers, Laoprom and colleagues examined genetic diversity and population structure of *O. viverrini*. A high level of genetic diversity was demonstrated for *O. viverrini* sampled in 5 localities [52]. Subsequent assessment of population structure by pairwise Fst analysis showed from low to middle level of inter-population differentiation [53]. In contrast, when multilocus enzyme electrophoresis was used to analyze genetic variation of *O. viverrini* from different geographical areas in Thailand and Lao PDR, high diversity between the isolates from different localities was observed. Thus, the authors speculated that *O. viverrini* did not represent a single species but rather comprised two cryptic species [54]. Unfortunately, no extensive research on the population genetics of *C. sinensis* has been published to date. Nevertheless, the genetic diversity study of *C. sinensis* population from the Russian Far East showed two levels of intraspecific variation for *ITS1* sequences [55].

Even though *O. felineus* is widely spread across Europe and Northern Asia, its intraspecific structure still remains poorly explored. To our knowledge, no analysis of its population genetic diversity has been done. Beer and German previously reported *O. felineus* to be heterogeneous within its distribution range [56]. A number of morphological features of eggs [7] or mature adults [57] were demonstrated to differ between geographically distant populations. Also, reciprocal cross-infection studies have identified local adaptation of parasite infectivity/host susceptibility for populations inhabiting different river systems [56]. As a result, three subspecies of *O. felineus* were proposed to exist: European, Siberian and Kazakh [7].

In the present work, we used a 260 bp-long marker fragment from the mitochondrial gene *cox1*, 573 bp-long fragment from *cox3* and a 481 bp-long fragment of a nuclear marker *ITS1* to gain insight into genetic diversity of *O. felineus* over its major distribution range. We also adopted this approach to address the question of its intraspecific population structure.

## Materials and Methods

### Sample Collection

**Figure 1:**
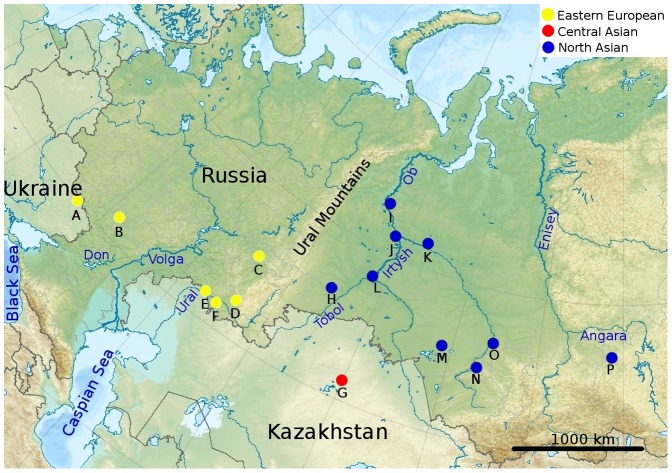
Geographical localities where the specimens were collected. Lettered names of collection sites correspond to those in Table 1.

*O. felineus* metacercariae were collected in different geographical localities in Eastern Europe (the Volga, the Don and the Ural river basins), Northern Asia (the Ob-Irtysh and the Yenisei basins) and Central Asia (the Nura-Sarysu basin) (Figure 1). Metacercariae were found in ides (*Leuciscus idus*), roaches (*Rutilus rutilus*), daces (*L. leuciscus baicalensis*) and rudds (*Scardinius erythrophthalmus*). We isolated DNA directly from 149 metacercariae. Species identity of the samples was established by comparing the sequencing reads with the reference sequences available (e.g. DQ456831– *O.felineus*, EU038154 - *Metorchis bilis* and EU483073 - *Pseudamphistomum truncatum*). Thus, 124 samples were classified as *O. felineus* and so they were used in this work. The remaining samples were from *P. truncatum* and *M. bilis* (Opisthorchiidae family). Also, adult worms (97 samples) were grown in the lab by infecting golden hamsters (*Mesocricetus auratus*) with metacercariae. One month after infection, adult worms were isolated from hamster livers. Species identity was morphologically assessed under the light microscope. Each adult worm was cut in 2–3 parts, one of which was used for DNA isolation. The remaining material was used as a back-up and stored in 70% ethanol at +4°C.

Altogether, 221 *O. felineus* individuals from 16 localities were used in our analysis (A–P, Figure 1 and Table 1).

**Table 1:** Geographical localities and numbers of individuals sequenced for *cox1* and *ITS1*.

Letter code	Origin of isolates (metacercaria): oblast/river/river basin	No of individuals sequenced			Genbank accession №		
		*cox1*	*ITS1*	*cox3*	*cox1*	*ITS1*	*cox3*
**Eastern Europe**							
A	Kharkiv^U^/Seversky Donets/Don	3	0	3	JX913340-42		KC188972-74
B	Voronezh^R^/Savala/Don	15	1	15	JN646337-41 JN646356 JN646628-35 JN646643	JX913423	KC188936-48 KC188967-68
C	Republic of Bashkortostan^R/^Belaya/Volga	14	9	11	JN646269-76 JN646583, KC619541-45	JX913393-401	KC188849-55, KC619537-40
D	Orenburg^R^/Ilek/Ural	12	0	12	JN646329-35 JN646623-27		KC188923-34
E	Orenburg^R^/Sakmara/Ural	1	0	1	JN646336		KC188935
F	Orenburg^R^/Ural/Ural	11	2	11	JN646322-28, JN646355, JN646620-22	JX913421-22	KC188913-22 KC188966
**Central Asia**							
G	Akmola Province^K^/Nura/Nura-Sarysu	29	26	20	JX913343-71	JX913428-53	KC188975-94
**Northern Asia**							
H	Kurgan^R^/Iset/Irtysh	6	0	7	JN646345-50		KC188957-63
I	Khanty-Mansi Autonomous Okrug^R^/Ob/Lower Ob	23	16	8	JN646253-60 JN646278-83 JN646293 JN646581-82 JN646596-601	JX913374-83, JX913403-07, JX913410	KC188847-48 KC188872-77
J	Khanty-Mansi Autonomous Okrug^R^/Ob/Lower Ob	10	0	7	JN646294-97, JN646344, JN646638-42		KC188878 KC188951-56
K	Khanty-Mansi Autonomous Okrug^R^/Ob/Lower Ob	5	0	6	JN646290-92 JN646594-95		KC188866-71
L	Tyumen^R^/Tobol/Irtysh	25	13	16	JN646262-68, JN646314-21, JN646610-19	JX913385-92, JX913416-20	KC188897-912
M	Novosibirsk^R^/Om/Ob-Irtysh	12	0	10	JN646289, JN646306-13, JN646352-54		KC188889-96 KC188964-65
N	Novosibirsk^R^/Ob/Upper Ob	21	4	12	JN646277 JN646284-88 JN646342-43 JN646351 JN646584-93 JN646636-37	JX913402, JX913408-09, JX913424	KC188856-65 KC188949-50
O	Tomsk^R^/Tom/Upper Ob	21	7	12	JN646250-52 JN646298-305 JN646579-80 JN646602-09	JX913372-73, JX913411-15	KC188845-46 KC188879-88
P	Irkutsk^R^/Biryusa/Yenisei	4	4	3	JN646261, JX913337-39	JX913425-27, JX913384	KC188969-71
**Total**		212	82	154			

U – Ukraine, K – Kazakhstan and R – Russia. Representative sequences of the central predominant haplotypes for our markers can be found in Genbank under the following accession numbers: *cox1* (JN646261), *cox3* (KC188845) and *ITS1* (JX913384).

### Ethics Statement

Territories where sample collection (fishing) took place were neither conservation areas nor private, nor otherwise protected, hence no fishing permits were required. The fish species collected are not considered endangered or rare, and fishing methods were in full compliance with the Federal Law N166-Ф3 of 20.12.2004 (ed. 18.07.2011) "Fishing and conservation of water bio-resources”.

This study was carried out in strict accordance with the recommendations in the Guide for the Care and Use of Laboratory Animals of the National Institutes of Health. The protocol was approved by the Committee on the Ethics of Animal Experiments of the Institute of Cytology and Genetics (Permit Number: 7 of 19.11.2011). Euthanasia was performed by decapitation, and all efforts were made to minimize suffering.

### Choice of DNA Markers

Three markers showing different levels of polymorphism were selected for our analysis.

*Cox1* is a marker routinely used in population genetic studies. *Cox3* is yet another michonodrial gene which is known to have increased frequency of SNPs relatively to *cox1*. [58]. Our pilot analysis of three closely related species, *O. felineus, C. sinensis* and *O. viverrini,* demonstrated that *cox3* is indeed more variable than *cox1*. This established *cox3*, similarly to *nad1*, as one of the highly variable mtDNA markers in liver flukes.

Compared to the mtDNA markers used, the nuclear *ITS1* sequence is much more conserved. The specific choice of *ITS1* rather than *ITS2* sequence was based on the fact that it is the only region of rDNA which is variable enough to discriminate polymorphisms within species (even though this polymorphism is quite low) [49], [55].

### DNA Extraction, PCR Amplification and Sequencing

DNA was isolated from *O. felineus* adults or metacercariae using standard phenol/chloroform extraction [59] followed by precipitation with an equal volume of 100% isopropanol. DNA was dissolved in TE buffer and stored at −70°C.

Vector NTI Advance 10 (Invitrogen) was used for primer design. These primers were universal for the *Opisthorchis* genus. We aligned Genbank sequences for the four species of interest, and focused on the primer regions that were the most conserved.

*Cox1* gene fragment was amplified using the primer pair *cox1*-F (5′-GGGTTTGGAATGATTAGTC) and *cox1*-R (5′-CACAGAGGCAGAAAGAACT), Tm –54°C; fragment of *ITS1* was amplified with *ITS1*-F (5'-GTCGTAACAAGGTTTCCGTA) and *ITS1*-R (5'-ACACGAGCCGAGTGATCC), Tm –59°C; the *cox3* fragment was amplified with *cox3*-F (5'-ATATTTATGGGGATTGTGAATT) and *cox3*-R (5'-AGCCCGAACTATAGACAACA), Tm –50°C.

Reactions contained 100 pg template DNA, 250 mkM dNTPs, 25 mkM dUTP, primers (200 nM each), 0.1 U UDG (Fermentas), 1.5 mM MgCl_2_, 1.5 U TrueStart Hot Start Taq DNA polymerase (Fermentas) and appropriate PCR buffer. Touch-down PCR was performed in a total volume of 20 mkl with the following thermocycling conditions: 37°C/5 min, 94°C/2 min, 10 cycles: 94°C/10 sec, Tm+10°C, decrement −1°C per cycle/20 sec, 72°C/40 sec, 20 cycles: 94°C/10 sec, Tm/20 sec, 72°C/40 sec followed with the final elongation step at 72°C for 5 minutes.

Amplified DNA fragments were Sanger-sequenced using BigDye terminator cycle sequencing kit (Applied Biosystems), according to the manufacturer’s specifications. Sequencing primers were the same as those used for PCR. To assure the accuracy of sequencing, the amplified fragments were sequenced from both forward and reverse primers. Sequencing products were run on ABI 3130XL Genetic Analyzer (Applied Biosystems). In some individuals only one or two marker sequences could be retrieved.

Chromatogram analysis and editing were done using Sequencing Analysis 5.2, Patch 2 (Applied Biosystems). All the sequences were deposited in Genbank, and their accession numbers are shown in Table 1.

### Data Analysis

The sequences of *cox1*, *cox3* and *ITS1* were aligned separately using Vector NTI Advance 10 (Invitrogen). Intrapopulation variation was summarized using standard statistics: number of haplotypes (H), number of segregating sites (S), haplotype diversity (Hd) and nucleotide diversity (π), were all calculated using the program DnaSP 4.5 [60]. Haplotype diversity represents the probability that two randomly sampled alleles are different, while nucleotide diversity is defined as the average number of nucleotide differences per site in pairwise comparisons among DNA sequences [61]. The significance of Hd and π was tested using 999 coalescent simulations [62].

Due to heteroplasmy mtDNA and partial homogenization and/or heterozygosity rDNA, several sequences (3 *cox1*, 4 *cox3* and 7 *ITS1*) had degenerate sites. In these cases, haplotypes were reconstructed using PHASE algorithms provided in DnaSP 4.5 [63].

The parsimony network was constructed using the median joining algorithm from Network 4.6 [64].

The degree of gene flow between populations from three geographical regions was estimated using pairwise Fst [65], calculated by the program Arlequin 3.1 [66].

To investigate hierarchical levels of population structure, the analysis of molecular variance (AMOVA) was performed, considering genetic distances between haplotypes and their frequencies among the defined populations, with statistical significance determined by 1000 permutations [67], using Arlequin 3.1. To perform this test, localities were grouped according to their geographical proximity. The Eastern European group is formed by the populations from the Don river basin (A–B), the Volga river basin (C) and the Ural river basin (D–F). Four populations constitute the Northern Asian group, namely: the Irtysh river basin (H–L), the lower Ob basin (I–K), the upper Ob basin (N and O) and the Om river (M) populations. The latter population formally belongs to the Irtysh river basin, yet it is geographically closer to the upper Ob river basin. Finally, the Central Asian group includes a single population (G). No AMOVA was done for the population (P) from Irkutsk oblast (N<10) (Figure 1, Table 1).

Neutrality tests were performed using Tajima’s D [68] and Fu’s Fs [69], which were calculated using DnaSP 4.5. The significance of both values was calculated from 999 simulated samples using a coalescent algorithm [62]. These tests are important to discriminate the sequences found in mutation-drift equilibrium from the sequences evolving under non-neutral conditions. Non- neutral conditions include directional or balancing selection, and demographic expansion or contraction.

A distribution of pairwise genetic differences (mismatch distribution) analysis [70] was conducted to estimate population dynamics using DnaSP 4.5. The raggedness (r) statistic was used to assess the smoothness of the observed mismatch distribution [71], and tested for the significant departure from unimodality. Finally, we also produced an estimate of τ, the date of population expansion (τ = 2 ut; t being the time in generations, and u being the mutation rate per sequence per generation).

## Results

### Genetic Diversity

All *O. felineus* populations studied here were combined into three geographically isolated groups: Northern Asian (Siberian, the Ob-Irtysh and the Yenisei river basins), Central Asian (Kazakh, the Nura-Sarysu basin, part of the endorheic Aral-Caspian basin) and East European (drainage basins of the rivers Volga, Don, and Ural lacking natural barriers). This grouping was based on the data of German and Beer on the existence of three *O. felineus* subspecies in these territories [7], [56].

We sequenced one nuclear marker, *ITS1* (481 bp), and two mitochondrial markers, *cox1* (260 bp) and *cox3* (573 bp). The diversity indices calculated from the nucleotide data sets are summarized in Table 2.

**Table 2:** Diversity indices calculated from the nucleotide data sets for *cox1*, *cox3* and *ITS1*.

Region	N			S			H			Hd			π		
	*cox1*	*ITS1*	*cox3*	*cox1*	*ITS1*	*cox3*	*cox1*	*ITS1*	*cox3*	*cox1*	*ITS1*	*cox3*	*cox1*	*ITS1*	*cox3*
Eastern Europe	56	12	53	2	1	18	3	2	14	0.07±0.03	0.08±0.08	0.62±0.05	(3±1)×10^−4^	(2±2) ×10^−4^	(18±3) ×10^−4^ *
Central Asia	29	26	20	3	3	4	4	4	5	0.20±0.07	0.31±0.01	0.43±0.09	(7±3) ×10^−4^	(7±2) ×10^−4^	(9±3) ×10^−4^
Northern Asia	127	44	81	16	3	33	17	4	29	0.38±0.04*	0.13±0.05	0.64±0.01*	(19±2) ×10^−4^ *	(3±1) ×10^−4^	(21±3) ×10^−4^ *
All	212	82	154	17	5	43	19	6	38	0.28±0.03*	0.18±0.04*	0.61±0.03*	(13±2) ×10^−4^ *	(4±1) ×10^−4^ *	(19±2) ×10^−4^ *

Abbreviations are number of isolates examined (N), segregating sites (S), number of haplotypes (H), haplotype diversity (Hd) and nucleotide diversity (π).

* - p<0.05.

In agreement with the expected degree of variability of the markers used, *ITS1* and *cox3* displayed the least and the highest genetic diversity, respectively. Surprisingly, the levels of nucleotide and haplotype diversity were very low across the whole territory studied (Hd = 0.28 (*cox1*), 0.61 (*cox3*) and 0.18 (*ITS1*); π = 0.0013 (*cox1*), 0.0019 (*cox3*) and 0.0004 (*ITS1*).

In order to control for possible introduced bias while culturing metacercariae in the non-natural definitive host (*Mesocricetus auratus*), we separately analyzed genetic diversity in the samples obtained directly from metacercariae and from all adult worms grown in the lab. No significant differences were found (Table S1). This suggests that there was no selective pressure of culturing *O. felineus* in lab animals on the parasite diversity (as observed with the markers used).

### Haplotype Networks

**Figure 2:**
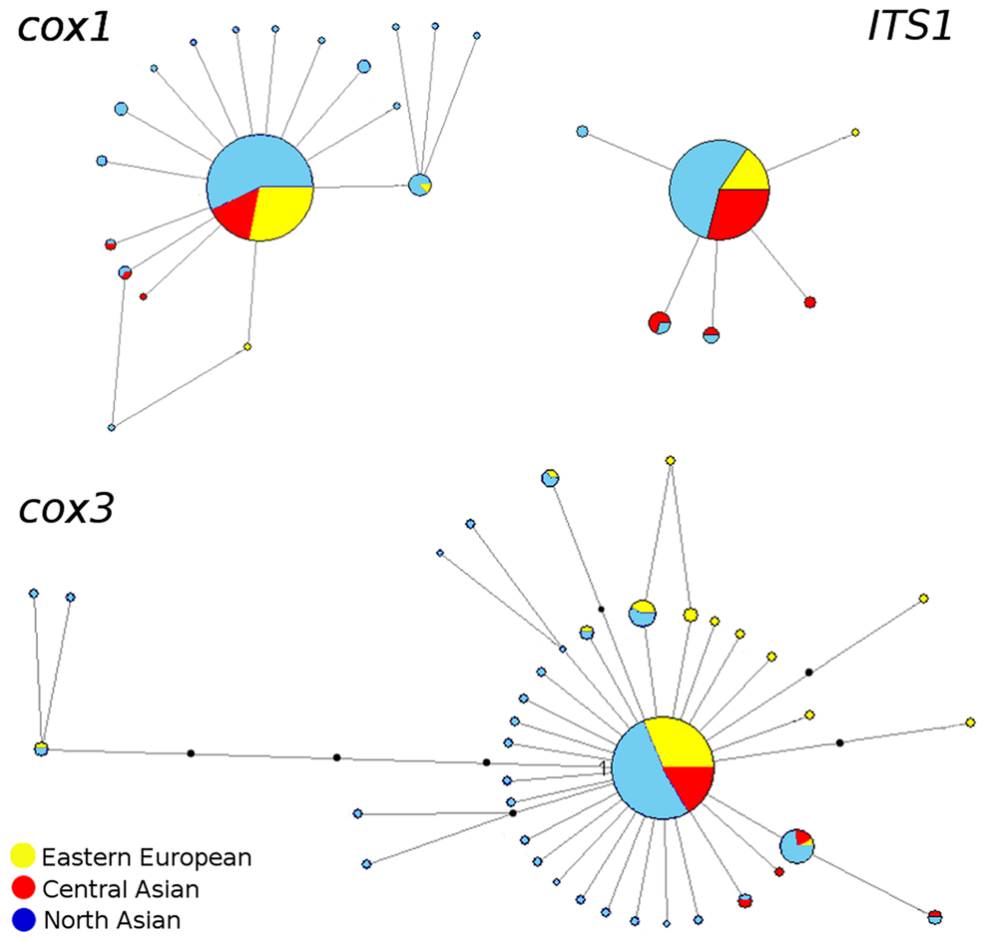
The statistical parsimony networks of *cox1, ITS1* and *cox3* haplotypes. The sizes of haplotypes are proportional to the number of samples. Small black circles indicate un-sampled or extinct haplotypes.

To establish the genealogical relationships between the haplotypes, we constructed a statistical parsimony network (Figure 2).

For each of the three markers used, the most common haplotype was present in all populations. Such haplotype was the most abundant and was identified in 84.7%, 61.7% and 88.1% of sampled individuals for *cox1*, *cox3* and *ITS1*, respectively, across the entire region studied. This main haplotype was also central to all other haplotypes, thereby forming a core of a star-like network.

Besides the common haplotypes, we identified 18 *cox1* haplotypes, of which 12 were unique (singlets), 37 *cox3* haplotypes (28 singlets) and 5 *ITS1* haplotypes (3 singlets). Most of the identified haplotypes were distinct from the central haplotype by one substitution (14– *cox1*, 5– *ITS1* and 25– *cox3*), or 2 substitutions (4– *cox1* and 9– *cox3*). In one case the haplotype diverged by as much as 4 SNPs from the central variant for *cox3*. Notably, this exceptional haplogroup was not restricted to a particular geographic location. We also found 6 non-singlet region-specific haplotypes. For *cox1* and *cox3*, there were 4 and 1 Northern Asia-specific haplotypes, respectively. One region-specific *cox3* haplotype was found in the Eastern Europe. All these haplotypes were exceedingly rare, i.e. in a given region only 2–3 individuals shared such variant.

### Genetic Differentiation of Populations

*Cox1-* and *cox3-*based analyses of molecular variance (AMOVA) failed to show considerable differences between separate populations and regions (Table 3). When performing comparisons at the group level (when all populations from each region are pooled into a single artificial population), we observed that over 98% of the total variance was due to intra-region differences. Fixation index (Фst) value was 0.011 for *cox1* and 0.013 for *cox3* (Table 3). Global AMOVA showed that inter-individual differences accounted for over 96% of the total variance. The obtained values of fixation indices (Фst and Фsc) were below 0.05 (Table 3), which is indicative of low genetic differentiation [72].

**Table 3:** Analysis of molecular variance (AMOVA) calculated from *cox1* and *cox3* nucleotide datasets.

Source of variation	*cox1*		*cox3*	
	% total variance	Fixation indices	% total variance	Fixation indices
**Group level**				
Among regions	1.28	Фst = 0.013*	1.09	Фst = 0.011*
Within populations	98.72		98.91	
**Global AMOVA**				
Among regions	0.06	Фct = 0.006Фsc = 0.015*Фst = 0.021*	−0.55	Фct = −0.005Фsc = 0.037*Фst = 0.032*
Among populations within groups	1.47		3.71	
Within populations	97.92		96.84	

* - p<0.05. Фst – genetic differences among populations, Фct – among groups, and Фsc – among populations within groups.

In several populations, there were too few *ITS1*-typed samples, so no AMOVA was performed for this marker.

Pairwise Fst values were estimated to assess genetic differentiation among 3 regions (Table 4). Inter-region Fst values were very low, ranging from 0.008 to 0.021.

**Table 4:** Pairwise fixation indices (Fst values) between *O. felineus* populations calculated from the nucleotide datasets for c*ox1*/*cox3*/*ITS1*.

	Northern Asia	Central Asia
Central Asia	0.014* **/**0.015*/0.020	
Eastern Europe	0.012* **/**0.008*/0.000	0.021* **/**0.012**/0.024

* - p<0.05,

** - 0.1>p>0.05.

As a result, low values of fixation indices indicate that the populations and regions are poorly genetically differentiated from one another.

### Demographic History

The results of Tajima's D test and Fu's F_S_ test shown in the Table 5 support the explosive growth pattern of the *O. felineus*. Tajima's D values were negative for all the markers used (−2.1 (*cox1*), −2.40 (*cox3*) and −1.55 (*ITS1*)), indicating an excess of rare nucleotide site variants compared to expected under a neutral model of evolution. Results of Fu's Fs test (which is based on the distribution of haplotypes) also show negative values (−29.70 (*cox1*), −48.83 (*cox3*) and −5.76 (*ITS1*)). This indicates an excess of rare haplotypes over what would be expected under neutrality. This could be due to population expansion, purifying selection or selective sweep. However, all the markers used here are known to be selectively neutral, therefore the effect observed should be best explained by population expansion.

**Table 5:** Neutrality indices calculated from the nucleotide datasets for *cox1*, *cox3* and *ITS1*.

Region	Tajima’s D			Fu’s Fs		
	*cox1*	*ITS1*	*cox3*	*cox1*	*ITS1*	*cox3*
Eastern Europe	−1.22	−1.16	−2.02*	−2.92	−1.03	−8.48*
Central Asia	−1.37	−1.04	−0.98	−2.86	−1.78	−1.92
Northern Asia	−2.05*	−1.42	−2.31*	−19.76*	−3.63*	−31.24*
All	−2.10*	−1.55*	−2.40*	−29.70*	−5.76*	−48.83*

* - p<0.05.

**Figure 3:**
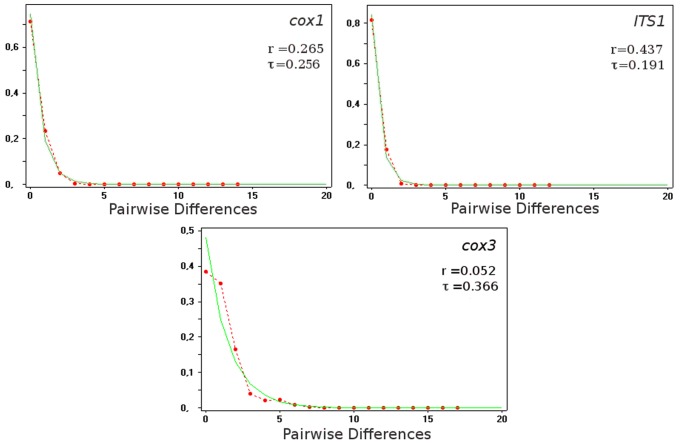
Mismatch distribution curve for *O. felineus* haplotypes. Expected values under expanding population model and observed values are shown as solid green and dashed red lines, respectively (r – ruggedness index).

To test the hypothesis of *O. felineus* population expansion, we calculated the distribution of pairwise nucleotide differences among individual haplotypes. The mismatch distribution for the species was clearly unimodal, and characterized with low ruggedness index r (which means the experimental distribution closely matches the expected values). Unimodal mismatch distributions are typical of expanding populations, whereas old populations of constant size are expected to be multimodal [73]. As it is shown on Figure 3, our data are consistent with the hypothesis of sudden expansion of *O. felineus*.

We also estimated τ (0.256 for *cox1*, 0.366 for *cox3* and 0.191 for *ITS1*), which is the date of growth or decline measured in units of mutational time, and is useful to estimate the onset of expansion [70].

Winter period ranges 5–8 months across *O. felineus’* distribution range, and most of cercariae leave the snails in June-July. It takes 2–2.5 months for the egg to develop inside the snail, whereas maturation of metacercaria in fish and adult worm in animals takes about 1 month [7]. Taking these numbers into account, we set the generation time of *O. felineus* at 1 year. In order to estimate the time of species expansion, we used the substitution rates previously obtained for *S. japonicum* and *S. indicum* (Schistosomidae, Trematoda): *ITS* rate being 0.8%/MY [74], *cox1*–2.5%/MY [75]. Based on these data, we believe that species demographic expansion started 21–25 kBP (thousands of years before present).

Thus, this analysis suggests that our species of interest has recently gone through a severe population bottleneck followed with sudden expansion (21–25 kBP) from a small group of founders with a very limited gene pool.

## Discussion

Nowadays it becomes increasingly important to study biodiversity of human parasites, given that many of such species are now observed to rapidly invade new territories and thereby pose an increasing epidemiological threat. Genetic diversity is one of the most important components contributing to species survival and ecosystem resilience. Knowledge of the genetic structure within and among populations of parasitic species combined with phylogeographic data can provide a deeper understanding of their ecological and evolutionary dynamics with practical implications for parasitosis control.

Nuclear and mtDNA markers are widely used to assess genetic relationships between individuals or groups of individuals. These markers are used to study the dynamics of genetic processes within populations, to describe the species population structure and to elucidate the phylogeographic history [76]. In the present work we used this approach to analyze for the first time the genetic diversity of the Siberian liver fluke *O. felineus* found in the largest river systems of Eastern Europe and Central and Northern Asia. We SNP-typed three DNA fragments from *ITS1*, *cox1*, and *cox3,* which have shown different substitution rates. The patterns of genetic structure and diversity obtained from these DNA markers were largely consistent.

### Low Genetic Diversity and Pronounced Star-like Haplotype Network

DNA markers similar to the ones used in our work have been extensively tested over the past ten years to establish the population structure and phylogeography of human-infecting trematodes from Europe [40], [41], Eastern and South-Eastern Asia [42], [51], [77], [78], Africa [27], [30] and America [41]. However, to our knowledge no such analysis of trematode species inhabiting both Northern Asia and Eastern Europe was done. When designing these experiments and sampling the *O. felineus* across its very large range, we expected to see complex intraspecific structure. However, our analysis revealed that three regions tested (Eastern European, Northern Asian and Central Asian) demonstrated very low values of genetic variation (Hd = 0.28 (*cox1*), 0.61 (*cox3*) and 0.18 (*ITS1*); π = 0.0013 (*cox1*), 0.0019 (*cox3*) and 0.0004 (*ITS1*). Nucleotide diversity was low because haplotypes typically differed by just a single nucleotide. Our estimates of genetic differentiation between the populations were based on the haplotypes frequencies and evolutionary distance between haplotypes. Fst<0.05 (0.008–0.021), as well as on AMOVA tests, were indicative of very low differences among populations. Haplotype networks displayed pronounced star-like structure with one major haplotype present in over 80% of cases for the relatively conserved *cox1* and *ITS1* and in over 60% of cases for the more variable *cox3*. The rest of the haplotypes were distinct from the major variant by one or two substitutions, and were predominantly singlets. Such peculiar pattern – low genetic diversity, star-like haplotype network with dominance of one major haplotype and many rare variants, combined with highly negative neutrality indices (Fu’s Fs and Tajima’s D reaching −29.70 and −2.10 (*cox1*), −48.83 and −2.40 (*cox3*) and −5.76 and −1.55 (*ITS1*) – are indicative of a recent severe bottleneck in the species population history, which was followed with rapid colonization and population expansion [79]. Clear predominance of the central haplotype suggests that this is the most recent common ancestral haplotype [80].

In addition to the recent severe bottleneck, the peculiar mating system in this species could also contribute to the decrease in genetic variability. Liver flukes are facultative hermaphrodites [7]. Even though some trematodes were demonstrated to prefer outcrossing to selfing [81]–[83], no such data are available for the *Opisthorchis* species. Selfers are expected to be less polymorphic than outcrossers [84], [85]. Nevertheless, genetic variability studies of natural populations of self-compatible hermaphrodites capable of both self-fertilization and outcrossing show low polymorphism and high inter-populational differentiation [86], [87]. Yet, in the case of *O. felineus*, differentiation both between and within populations was low.

Besides being hermaphroditic, trematodes display obligate alternation between sexual and clonal reproduction during their complex life cycle. Although modeling has suggested certain changes in the population genetics of such parasites [88], [89], unfortunately, very little is known about gene dynamics throughout the trematode life cycle.

Studies of other Eurasian trematode species with similar reproductive cycle reported significantly higher genetic diversity [39]–[41], [50], [55]. Notably, European *F. hepatica* populations showed extensive genetic mtDNA diversity even in local studies (Ireland and the Netherlands) [37], [38]. Partial sequences of mitochondrial genes *nad1* and *cox1* revealed 4.1 and 2.3% sequence variation, respectively, in flukes sampled across Eastern Europe [40]. As for other *Opisthorchis* species, *O. viverrini* populations displayed a striking Hd = 0.88 (*nad1*) just in the Lower Mekong River basin. Of the 19 haplotypes found in this population, there were at least three major haplotypes that were considerably distinct from each other [50]. Two distinct phylogenetic *ITS1* clades were found in an other *Opisthorchis* species, *C. sinensis*, in its Russian Far East population [55].

Taken together, these data indicate that the peculiar mode of *O. felineus* reproduction is not the main factor contributing to its extraordinary low genetic variability. Thus, we suggest that the low genetic diversity of *O. felineus* is best explained by some sort of environmental impact (such as severe climatic changes) which caused the effective population size to shrink. Whatever the case, as the population was undergoing expansion after the bottleneck, its low genetic variability should have largely relied on higher levels of genetic drift due to the stage of very active asexual reproduction in the life cycle of this species.

### Phylogeography of *O. felineus*

To gain deeper insight into the possible causes of low genetic variability in our species of interest, a phylogeographic approach can be very useful. Climatic oscillations of the Pleistocene are often invoked as major events that have shaped the geographic and genetic structure of current species via past range fragmentation, population contraction and recolonisation [90].

Our mismatch distribution test clearly suggested that *O. felineus* is presently undergoing demographic expansion, which started relatively recently, approximately 21–25 kBP. We estimated that *O. felineus* split from the last common ancestor with *C. sinensis* about 3 MYA (an estimate based on mtDNA sequence, data not shown). Back then, Northern Asia had relatively mild climate, and in Siberia, Sino-Indian species of flora and fauna were widely distributed [91]. Later palaeogeography records suggest that 2.8 MYA a “cryogenic epoch” started in Western and Eastern Siberia, which was characterized with much colder climate and severe geocryological conditions [92]. During the later Quaternary period, northern parts of Eurasia underwent continuous glacial expansions and marine transgressions. According to the accepted chronological data, the last glaciation in Northern Eurasia started about 80 kBP and was characterized with interstadial conditions around 50–30 kBP transitioning to a much colder and drier environment. This occured at the culmination of the Last Glacial maximum (LGM) at 23–15 kBP, with the beginning of warming around 15–10 kBP [93], [94]. Given that our molecular dating is quite approximate, we cannot unambiguously determine whether the Siberian liver fluke started its demographic expansion at the interstadial period before LGM or soon after LGM. Climate warming and active melting of ice across the territory of Northern Eurasia resulted in massive rerouting of the drainage [95]. In all likelihood, this could be the perfect time for the geographical expansion of a single surviving ancestral population of *O. felineus* across the draining territories. The major reason underlying the severe bottleneck that Siberian liver fluke population went through is harsh environment in the Quaternary combined with overall low infestation rate (<1%) [7] of the first intermediate host, a fresh-water snail. Much like present time, distribution and availability of such hosts served as the main limiting factor in the completion of the full life cycle of liver flukes on their endemic territories.

It would be interesting to pinpoint the location of *O. felineus* refugium and to trace its migration routes. Unfortunately, the species’ low genetic diversity across the territory studied suggests the refugium was located elsewhere. Where would the most likely location of such refugium be? There is a line of fossil evidence suggesting that during the climate fluctuations and landscape changes in Pleistocene, Bithyniidae snails went essentially extinct in the present-day Ukraine and European part of Russia [96]. Yet, they appear to have persisted in Kazakhstan and southern parts of Western Siberia [7]. There are several refugiums known for terrestrial animals and arcto-tertiary flora in these regions [97]. However, during LGM the water bodies were completely frozen in wintertime in such refugiums [94], [98], so the water refugiums for the snails, the first intermediate hosts, must have been located more to the south. Modern modeling suggests that about 80 kBP the growth of the Barents–Kara Sea Ice Sheet led to the formation of an ice-dammed lake on the territory of Western Siberia and to re-routing of the rivers Ob and Yenisei into the Aral Sea [95]. Back then, the Aral Sea was part of the Ponto-Caspian basin, an essential refugium for the European aquatic fauna [99], [100]. Alternatively, the refugium could also have been located down the Pleistocene line of drainage of Siberian rivers that ran along the Turgai valley on the north-west of Kazakhstan, where the lake system of early Pleistocene formation is still present. In this region, Pliocene and Pleistocene relict flora can be found [101] indicative of relatively mild climate during LGM. Clearly, in order to determine the real location of refugium from which the ancestral population expanded to the present range, a wider analysis of various localities across the Central Asia and Europe is needed.

*O. felineus* hosts include fresh-water and terrestrial species of animals, therefore this parasite could spread over the new territories by different ways. Obviously this has contributed to the rapid geographic expansion of the species. The land route of *O. felineus* colonization could occur upon completion of LGM via fish-eating animals, whose long-distance migrations upon post-glacial climate changes were reported [102]. Also, beginning 9–10 kBP, European and Siberian territories have seen numerous human migrations, including re-colonization of Eastern and North-Eastern Europe by aboriginal West Siberian tribes throughout the mid-Holocene [103], [104]. Because of high susceptibility to the parasite and due to their high mobility, humans could have significantly contributed to the dissemination of liver flukes.

### Lack of Population Genetic Structure in *O. felineus*

Using standard DNA markers, we have shown very low levels of genetic differentiation between geographically distinct Europian and Asian populations of the Siberian liver fluke. In contrast to free-living species, there are far more factors that influence the genetic structure of parasites due to their complex life cycle and to the contribution of host biology. Trematodes have been previously reported to have less structured populations and lower genetic diversity as compared to snails, their first intermediate hosts [105]–[109]. It is also known that population genetic structure of parasites with complex life cycles is dependent on the mobility of the definitive hosts [107], [110]. It was suggested that the definitive host, which is often the most mobile of the host species, commonly leads to a high gene flow among local parasite populations, thereby lowering their differentiation [111]. Thus, the homogenization of population structure observed in our analysis could also result from potentially high gene flow between populations, which accompanied active migrations of definitive hosts, including humans, during the Holocene. However we tend to believe that the gene flow mediated by definitive hosts is not strong enough to consider the all populations as a single panmictic meta-population for the following reasons. First, high gene flow normally implies low local host/parasite adaptation, however this was not the case as shown by cross-infection studies [7], [56]. Second, population genetic analysis of a sister species *O. viverrini* demonstrated limited gene flow between the nearby regions [53]. Finally, it was shown that despite the high mobility of definitive hosts (cattle and humans), genetic differentiation between populations of yet another Eurasian trematode species, *F. hepatica*, dating back to the Quaternary events is still very well-discernible [39], [41]. Thus, it is not the ongoing gene flow among populations, but rather expansion of a single ancestral population over the present range that has likely shaped the observed uniform genetic make-up of *O. felineus*.

Absence of intra-species structure is in good agreement with short post-glaciation history of *O. felineus* and re-colonization of the range from a single source population. Yet, earlier studies proposed that *O. felineus* is a composite of several lineages, subspecies or cryptic species inhabiting the territory being analyzed. These proposals were based on the noticeable morphological differences among the isolates collected from different localities [57], [112]. Additionally, limited compatibility between the parasites and snails from geographically distant populations has been reported, which argued in favor of existence of several *O. felineus* subspecies [7], [56].

The markers used in our analysis are sensitive enough to track speciation process and to discriminate valid species [49], [113]. With these markers in hand, we demonstrated high levels of genetic homogeneity of *O. felineus* which prevented us from singling out any cryptic species or subspecies. Clearly, this result conflicts with the morphology findings. Much like other trematode researchers, we favor molecular data over morphological classifications [49], [114]. Trematodes have been notoriously known for their high overall morphological similarity as well as considerable plasticity of adult forms [115]–[117]. Indeed, in laboratory experiments, liver flukes showed high variability of morphological traits, depending on the specific culturing conditions, such as duration of infection and definitive host species [117].

Limited compatibility of parasites and snails from different geographical localities can not be easily disregarded and implies some intraspecies diversity was necessary for successful host/parasite adaptation. Such local adaptation relies on the interplay of highly variable proteins of hosts and parasites [118]. With this in mind, underlying genetic changes in the population can occur quite rapidly. As was shown for *S. mansoni*, as little as 3 generations were enough to form host-parasite compatibility in lab experiments [119]. The markers that we used in our analysis can not detect such quick evolutionary dynamics. However, taking into account the limited host/parasite compatibility between geographically distinct natural populations, large-scale population genetic analysis employing highly variable DNA markers or RAD-seq for both parasites and snail hosts should be of great interest and may also shed light on the post-glaciation migratory routes of *O. felineus*.

## Conclusions

The first population genetic analysis of Siberian liver fluke *O. felineus* revealed its uniquely low genetic diversity across a geographically enormous range. We suggest that severe geoclimatic events during the Quaternary epoch led to virtually complete extinction of this species. Upon completion of the last glaciation, *O. felineus* underwent rapid expansion from a single ancestral population. In fact, this recovery of species could have occurred from a very small ancestral population, due to the ability of this parasite to reproduce clonally. Importantly, despite the recent bottleneck episode, *O. felineus* is presently a widely-distributed species. Therefore, high dispersal potential of this trematode species should be taken into account in parasite-control programs.

## Supporting Information

Table S1 **Comparison of diversity indices of samples isolated from adult worms and metacercariae.**
(DOC)
