# Supplementary material for: Low Genetic Diversity in Wide-Spread Eurasian Liver Fluke Opisthorchis felineus Suggests Special Demographic History of This Trematode Species
Source: PLoS One. 2013 Apr 25;8(4):e62453. doi: 10.1371/journal.pone.0062453 (PMC3636034; doi:10.1371/journal.pone.0062453)
Supplement: Table S1 — Comparison of diversity indices of samples isolated from adult worms and metacercariae. (DOC) [file pone.0062453.s001.doc]

Table S1. Comparison of diversity indices of samples isolated from adult worms and metacercariae.

| Source | N | | | S | | | H | | | Hd | | | π | | |
| --- | --- | --- | --- | --- | --- | --- | --- | --- | --- | --- | --- | --- | --- | --- | --- |
|  | *cox1* | *ITS1* | *cox3* | *cox1* | *ITS1* | *cox3* | *cox1* | *ITS1* | *cox3* | *cox1* | *ITS1* | *cox3* | *cox1* | *ITS1* | *cox3* |
| Adult worms | 95 | 58 | 58 | 10 | 4 | 20 | 10 | 5 | 15 | 0.23±0.05* | 0.15±0.05 | 0.56±0.06* | (12±2)х10^-4^* | (3±1)х10^-4^* | (17±3)х10^-4^* |
| Metacercariae | 117 | 24 | 96 | 13 | 2 | 35 | 14 | 3 | 30 | 0.32±0.04* | 0.26±0.08 | 0.64±0.04 | (14±2)х10^-4^* | (6±2)х10^-4^ | (20±2)х10^-4^* |
| All | 212 | 82 | 154 | 17 | 5 | 43 | 19 | 6 | 38 | 0.28±0.03* | 0.18±0.04* | 0.61±0.03* | (13±2)х10^-4^* | (4±1)х10^-4^* | (19±2)х10^-4^* |

**Abbreviations are number of isolates examined (N), segregating sites (S), number of haplotypes (H), haplotype diversity (Hd) and nucleotide diversity (π). * - p<0.05**
